# Machine learning for early prediction of in‐hospital cardiac arrest in patients with acute coronary syndromes

**DOI:** 10.1002/clc.23541

**Published:** 2021-02-14

**Authors:** Ting Ting Wu, Xiu Quan Lin, Yan Mu, Hong Li, Yang Song Guo

**Affiliations:** ^1^ The School of Nursing Fujian Medical University Fujian China; ^2^ Department for Chronic and Noncommunicable Fujian Provincial Center for Disease Control and Prevention Fujian China; ^3^ Department of Nursing Shengli Clinical Medical College of Fujian Medical University, Fujian Provincial Hospital Fujian China; ^4^ Department of Cardiovascular Medicine Shengli Clinical Medical College of Fujian Medical University, Fujian Provincial Hospital Fujian China

**Keywords:** cardiac arrest, machine learning, prediction, XGBoost

## Abstract

**Background:**

Previous studies have used machine leaning to predict clinical deterioration to improve outcome prediction. However, no study has used machine learning to predict cardiac arrest in patients with acute coronary syndrome (ACS). Algorithms are required to generate high‐performance models for predicting cardiac arrest in ACS patients with multivariate features.

**Hypothesis:**

Machine learning algorithms will significantly improve outcome prediction of cardiac arrest in ACS patients.

**Methods:**

This retrospective cohort study reviewed 166 ACS patients who had in‐hospital cardiac arrest. Eight machine learning algorithms were trained using multivariate clinical features obtained 24 h prior to the onset of cardiac arrest. All machine learning models were compared to each other and to existing risk prediction scores (Global Registry of Acute Coronary Events, National Early Warning Score, and Modified Early Warning Score) using the area under the receiver operating characteristic curve (AUROC).

**Results:**

The XGBoost model provided the best performance with regard to AUC (0.958 [95%CI: 0.938–0.978]), accuracy (88.9%), sensitivity (73%), negative predictive value (89%), and F1 score (80%) compared with other machine learning models. The K‐nearest neighbor model generated the best specificity (99.3%) and positive predictive value (93.8%) metrics, but had low and unacceptable values for sensitivity and AUC. Most, but not all, machine learning models outperformed the existing risk prediction scores.

**Conclusions:**

The XGBoost model, which was generated based on a machine learning algorithm, has high potential to be used to predict cardiac arrest in ACS patients. This proposed model significantly improves outcome prediction compared to existing risk prediction scores.

**Abbreviations**
ACS: acute coronary syndrome
GRACE: Global Registry of Acute Coronary Events
IHCA: in‐hospital cardiac arrest
KNN: K‐nearest neighbor
MEWS: Modified Early Warning Score
NEWS: National Early Warning Score
NPV: negative predictive value
PPV: positive predictive value
SVM: support vector machine

## 1 INTRODUCTION

Cardiac arrest is a life‐threatening event and a leading cause of mortality globally.^1^ Accurate identification of high‐risk patients, adequate preparation, and prompt initiation of clinical management are paramount steps for successful cardiac arrest resuscitation.^2^ Among these steps, accurately identifying patients who are at high‐risk of suffering cardiac arrest is a primary strategy, and various studies have been conducted to predict the risk of cardiac arrest.^3^, ^4^, ^5^ Traditional studies commonly use standard statistical methods, such as regression‐based stepwise analysis to identify group‐level differences, and often include a limited number of variables.^3^, ^4^, ^5^ In contrast, machine learning begins with observations on an individual level, automatically searches multivariate data, extracts reliable outcome predictions, and ultimately generates reliable models.^6^

Machine learning has been regarded as an indispensable method for handling complex problems in science, especially in biomedical and astronomical research.^7^, ^8^ Recently, machine learning has emerged as a promising tool in the field of medicine, as well. With advances in algorithm technology, it is now possible to identify highly relevant features and discover new ways to utilize medical signals to improve the accuracy and functionality of prediction models to solve medical issues. Compared to prediction models of cardiac arrest generated using traditional methods such as regression method analysis or expert opinion, machine learning can achieve a better performance in many cases.^9^, ^10^, ^11^, ^12^, ^13^ In addition, current risk scores generated using traditional methods have limitations in clinical use due to their poor performance, low sensitivity, and/or a high false‐alarm rate.^14^

Despite the potential benefit of machine learning algorithms, several factors need to be taken into account when building a feasible algorithm for predicting cardiac arrest. First, in recent years, some studies extracted clinical features based on only on a patient's vital signs to generate an early warning system to predict cardiac arrest.^12^, ^14^ However, many of these attributes were not valuable and insufficient for stratifying the onset of cardiac arrest.^15^ Second, several studies have used machine learning to predict cardiac arrest in pediatric,^13^ septic,^9^ and ward patients,^12^ however, no previous research has used machine learning to predict cardiac arrest in acute coronary syndrome (ACS) patients. Finally, although some models derived from machine learning algorithms can accurately predict cardiac arrest, most studies failed to generate a visualization risk score. XGBoost is an ensemble algorithm based on gradient boosted trees that has an appreciable reputation with regard to overcoming numerous machine learning challenges, but has been seldom used for predicting cardiac arrest.

In the present study, we aimed to extract multivariate clinical features of ACS patients recorded in a database registry, and used various machine learning algorithms to develop several models that had appreciable performance for predicting cardiac arrest in ACS patients. We also endeavored to visualize the machine learning model, which we proposed in order to provide face validity for clinicians and researchers who are interested in implementing this technique. Additionally, we compared the predictability of machine learning with well‐known existing risk prediction models for ACS patients, such as Global Registry of Acute Coronary Events (GRACE),^16^ National Early Warning Score (NEWS),^17^ and Modified Early Warning Score (MEWS).^18^

## 2 METHODS

### 2.1 Study setting

This was a retrospective, observational study. All adult patients were diagnosed with ACS and hospitalized in the wards and intensive care units (ICUs) at three tertiary hospitals in Fujian province, China, between January 2012 and December 2016. These three hospitals had approximately 1200, 2500, and 1900 annual admissions of ACS patients, respectively. All nurses and physicians had successfully passed Advanced Cardiac Life Support training to ensure their ability to resuscitate patients. The study protocol was approved by the Fujian Provincial Hospital Institutional Review Board, and a waiver of informed consent was granted based on the minimal harm and general impracticability.

### 2.2 Populations

A total of 21 337 ACS patients documented in the registry between January 2012 and December 2016 were initially screened. In‐hospital cardiac arrest was defined as a loss of pulse due to pulseless ventricular tachycardia or ventricular fibrillation, pulseless electrical activity, or asystole. In this study, we defined cardiac arrest as the start of cardiopulmonary resuscitation and/or defibrillation. All cardiac arrest events were reviewed by a manual chart to ensure data quality. Patients who met the following criteria were included in the case group: (1) age ≥ 18 years, and (2) diagnosis with unstable angina, acute ST‐segment elevation myocardial infarction, or acute non‐ST segment elevation. Patients who had one of the following were excluded from this study: (1) a do not resuscitate order; (2) prior out‐of‐hospital cardiac arrest and ongoing resuscitation at admission; (3) cardiac arrest that had occurred within 24 h after admission or during an operation; (4) secondary multiple organ dysfunction syndrome; and (5) missing data. For patients with more than one cardiac arrest during the same period of hospitalization, only the first cardiac arrest was included in this study. The control group included patients admitted with ACS who did not experience a cardiac arrest during the 3‐year study period. Patients in the control group were randomly selected through the database, and the control group was roughly three times larger than the case group in order to satisfy modeling algorithm assumptions. Inclusion criteria for the control group were similar to the case group except that control patients did not have cardiac arrest during hospitalization. Control patients were excluded if they had been discharged “against advice” or had missing data.

After application of the inclusion and exclusion criteria, a total of 166 patients with cardiac arrest were included in the case group, and a total of 521 patients without cardiac arrest were included in the control group.

### 2.3 Candidate features

Two groups of features, which were used as potential predictor variables, were obtained from the electronic health record. One group of features, including age, gender, history of smoking, history of drinking, ACS type, culprit artery, and comorbidities, was registered at the time of admission and did not change during the hospitalization. The other group of features, including laboratory features, Killip classification, vital signs, mental status, the number of days prior to the occurrence of cardiac arrest, imaging and electrocardiogram examinations, were recorded 24 h preceding cardiac arrest (for patients who did not experience cardiac arrest, a random 24 h period was selected to collect data). Finally, a total of 45 features were selected as candidate features.

### 2.4 Probability analysis process

#### 2.4.1 Data preparation

**FIGURE 1:**
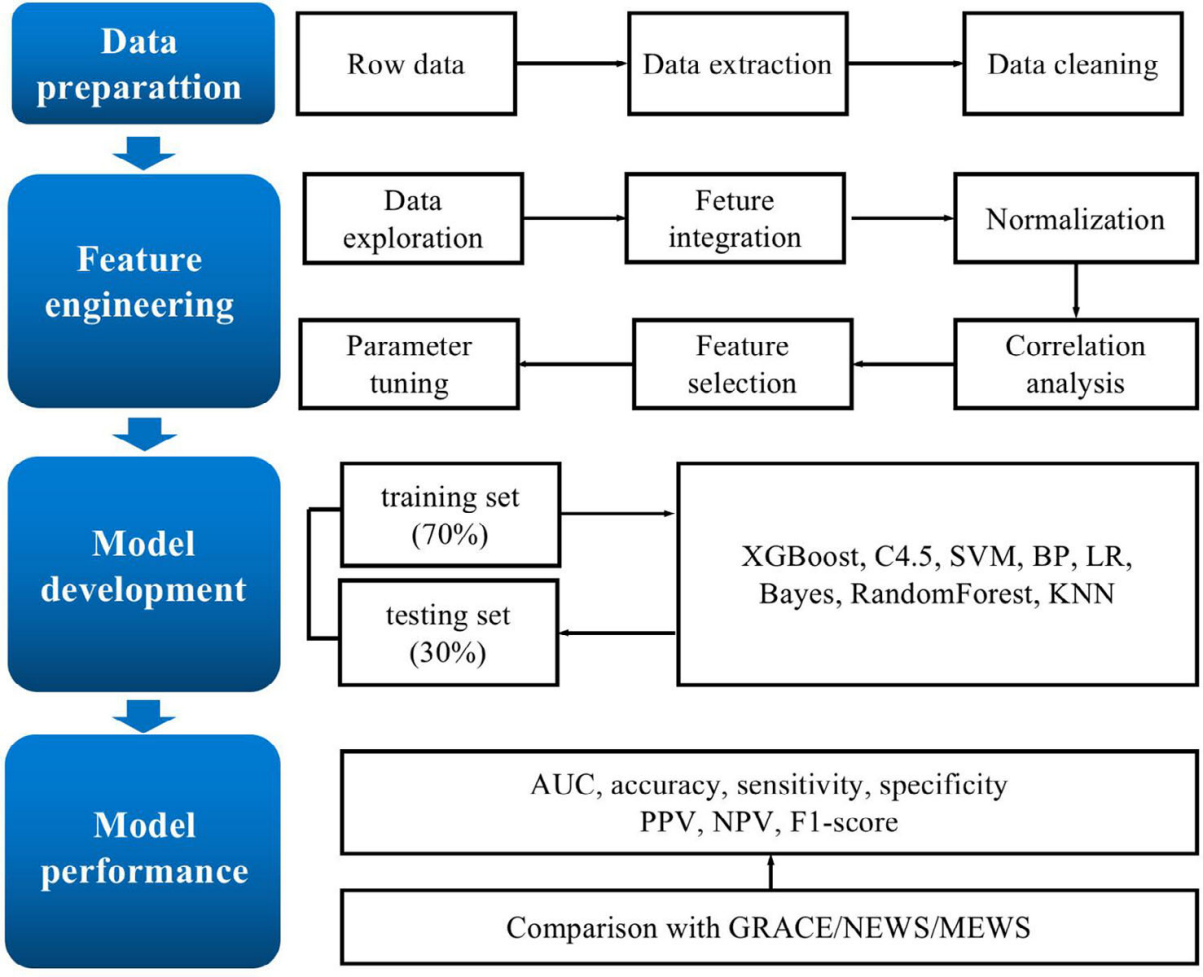
Probability analysis flow chart. SVM, support vector machine; BP, back propagation neural network; LR, logistic regression; KNN, K‐nearest neighbor; PPV, positive predictive value; NPV, negative predictive value

The flow chart of the probability analysis is shown in Figure 1. We adopted the imputation and discretization methods to clean data and deal with noise, missing values, and outliers. We discarded variables with 50% or more missing values. Some machine learning has decreased accuracy in unbalanced data,^19^ as observed in our study, so we matched positive samples (event group) to randomly selected negative samples (non‐event group) for the training model.

#### 2.4.2 Feature engineering

We normalized the data and target values, which were locked at 0–1. A total of 45 candidate features, which described the risk of cardiac arrest, were collected in this study. If all features were present, it would not only increase the computational burden, but also make the calculation very difficult. Therefore, in this study, correlation analyses were applied for feature selection to minimize the number of features. All features with a *p* < .01 and correlation coefficient > 0 were determined to be associated with cardiac arrest. The XGboost algorithm provided the important score of each feature.

#### 2.4.3 Model development

The dataset was randomly split into two sets: the training set (70% of participants) and the testing set (30% of participants). Eight machine learning algorithms were employed to develop cardiac arrest prediction models. XGBoost is a machine learning algorithm that assembles weak prediction models (typically decision trees) to yield a satisfactory predictive results.^20^ In the classification tree, the inside nodes represent values for an attribute test and the leaf nodes with scores represent a decision. Seven state‐of‐the‐art algorithms including C4.5, random forest, logistic regression, support vector machine (SVM), back propagation (BP) neural network, Bayes, and K‐nearest neighbor (KNN), were used to construct a model for early prediction of cardiac arrest.

#### 2.4.4 Model comparisons

Model discrimination was assessed using the area under the receiver‐operator curve (AUC). Six other performance metrics, including sensitivity, specificity, positive predictive value (PPV), negative predictive value (NPV), accuracy, and F1 score, were calculated to evaluate the performance of the model for the testing set. To evaluate the superiority of prediction capability of machine learning models, we compared those models with three existing model systems ‐ GRACE, NEWS, and MEWS ‐ using the same patient group.

#### 2.4.5 Statistical analysis

Continuous variables are expressed as mean ± standard deviation or median with an interquartile range, and categorical variables are expressed as frequency and percentage. Patient characteristics were compared using t‐tests, Wilcoxon rank‐sum tests, and *χ* ^2^ tests where appropriate. All *p* values were two tailed, and *p* < .05 was considered statistically significant. Python 3.7 was used for all statistical analysis.

## 3 RESULTS

### 3.1 Patient characteristics

**FIGURE 2:**
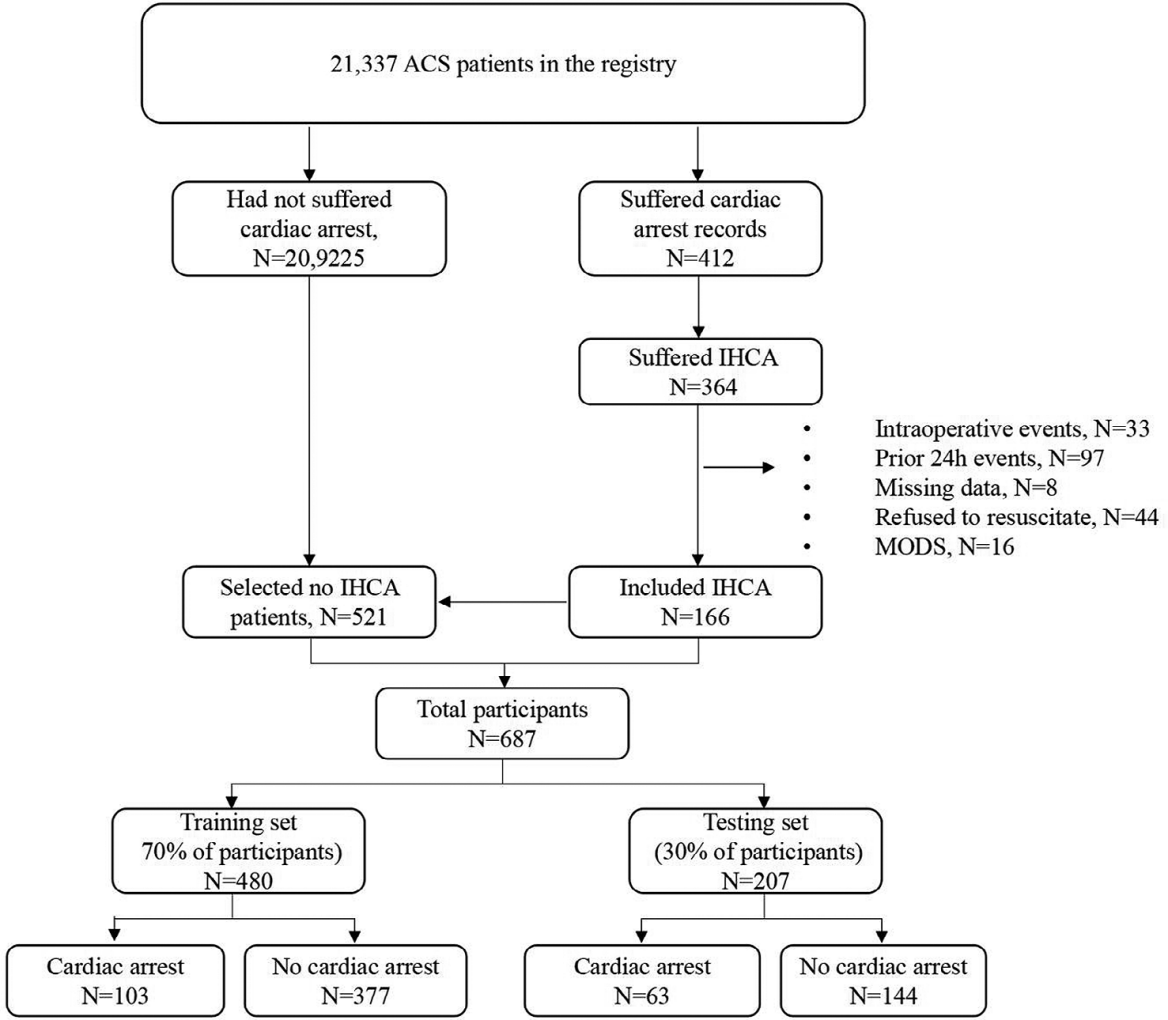
Enrollment of patients for the training and testing sets. ACS, acute coronary syndrome; IHCA, in‐hospital cardiac arrest; MODS, multiple organ dysfunction syndrome

This study consisted of 166 patients with cardiac arrest in the case group, and 521 patients without cardiac arrest in the control group. We randomly assigned 480 of these participants (70%) to the training set, and the remaining 207 participants (30%) to the testing set (Figure 2). Patient characteristics in the training and testing sets are listed in Table 1, and there were no significant differences between the control and case groups in either of the datasets with regard to patient characteristics.

**TABLE 1:** Characteristics of training and testing sets

Features	Training set *N*(%), *N* = 480 (70%)	Testing set *N*(%), *N* = 207 (30%)	*p* value
Cardiac arrest	103 (21.5)	63 (30.4)	.012
Male	378 (78.8)	150 (72.5)	.073
Drinking	78 (16.2)	20 (9.7)	.070
Smoking	239 (49.8)	91 (44.0)	.160
Killip classification			
І	156 (32.6)	67 (32.4)	.711
II	130 (27.1)	64 (30.9)	
III	88 (18.3)	35 (16.9)	
IV	106 (22.1)	41 (19.8)	
Fatal arrhythmia			
Atrial arrhythmia	32 (6.7)	11 (5.3)	.282
Borderline arrhythmia	26 (5.4)	5 (2.4)	
Ventricular arrhythmia	69 (14.4)	34 (16.4)	
Chest pain	145 (30.2)	72 (34.8)	.237
Age, year	68 (58, 77)	69 (59, 77) 59 77	.591
Temperature	36.5 (36.4, 36.6)	36.5 (36.4, 36.7)	.567
Heart rate	76 (66.25, 90.75)	78 (68, 92)	.143
Respiratory rate	20 (19, 2 0)	20 (18, 20)	.406
SBP	114 (103, 130)	110 (101, 124)	.044
DBP	66 (59, 75)	65 (59, 73)	.454
Mean arterial pressure	80 (74, 93)	80 (74, 90)	.118
Pulse pressure index	41.2 (36.4, 47.9) 36.4 47.9	41.2 (35.9, 46.6)	.405
Length of days prior to CA, day	2 (1, 3)	2 (1, 4)	.075
cTnI, micro/L	2.01 (0.28, 10)	2.01 (0.21, 13.7)	.607
Hematocrit	0.375 (0.333, 0.416)	0.372 (0.333, 0.415)	.475
Platelet count,×10^9^/L	212(169.25257)	212(170 260)	.989
BNP, pg/L	1737(5 055 082)	1737(5 645 652)	.702
Bilirubin, μmol/L	13.01(9.76, 17.02)	13.01(10.03, 16.5)	.481
WBC, ×10^9^/L	9.3(7.1, 12.2)	9.4(7.2, 12.1)	.850
Blood glucose,mmol/L	6.3(5.3, 8.25)	6.3(5.24, 7.57)	.601
Scr, μmol/L	87(70.35117)	87(70.9112)	.318
BUN, mmol/L	5.98(4.5, 8.43)	5.96(4.1, 8.8)	.296

Abbreviations: BNP, brain natriuretic peptide; BUN, blood urea nitrogen; cTnI, cardiac troponin I; DBP, diastolic blood pressure; SBP, systolic blood pressure; Scr, serum creatinine; WBC, white blood cell.

### 3.2 Feature selection

**FIGURE 3:**
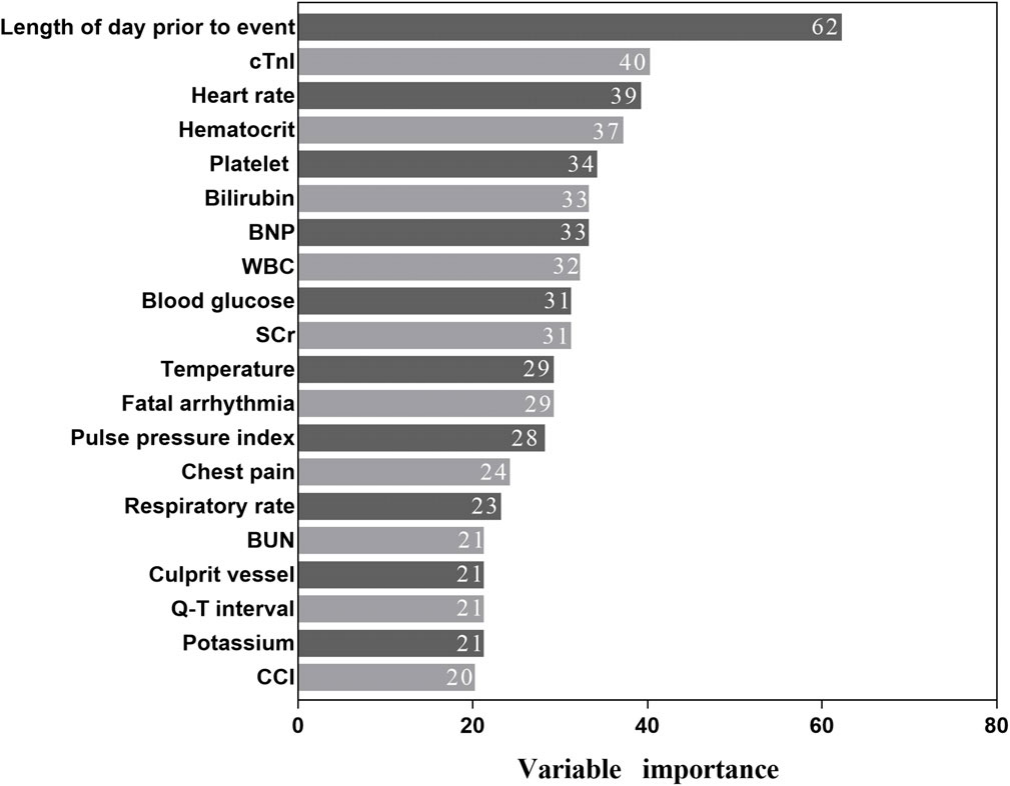
Importance of the candidate features. cTnI, cardiac troponin I; BNP, brain natriuretic peptide; WBC, white blood cell; SCr, serum creatinine; BUN, blood urea nitrogen; CCI:charlson comorbidities index

After feature selection using correlation analyses, we reduced the number of features from 45 to 20. Machine learning models were then developed based on the different combinations of these 20 features. Among these 20 features, we found that the number of days prior to the occurrence of cardiac arrest, cardiac troponin I, heart rate, and hematocrit were the four most important predictor features (Figure 3).

### 3.3 Evaluation of machine learning models

Our model discrimination analysis showed that the XGboost model was the most accurate (AUC: 0.958 [95%CI: 0.938–0.978]), followed by the C4.5 model (AUC: 0.784 [95%CI: 0.73–0.836]), the logistic regression model (AUC: 0.769 [95%CI: 0.719–0.825]), the random forest model (AUC: 0.747 [95%CI: 0.695–0.8]), the Bayes model (AUC: 0.734 [95%CI: 0.678–0.785]), the SVM model (AUC: 0.723 [95%CI: 0.671–0.773]), the BP neural network model (AUC: 0.723 [95%CI: 0.670–0.779]), and the KNN model (AUC: 0.616 [95%CI: 0.751–0.658]) (Figure S1A). These algorithms produced a specificity value greater than 89.6%, a sensitivity value ranging from 23.8% to 73%, and an F1‐score ranging from 38% to 80%, respectively. In general, the XGBoost algorithm provided the best overall performance regarding the AUC, accuracy, sensitivity, NPV, and F1 score compared with the other algorithms, and the KNN algorithm generated the best performance for the specificity and PPV metrics (Table 2). However, the KNN algorithm produced low and unacceptable sensitivity and AUC (Table 2).

**TABLE 2:** Cardiac arrest prediction performance

	AUC	95%*CI*	accuracy	specificity	sensitivity	NPV/precision	PPV	F1 score
XGBoost	0.958	0.937–0.978	0.889	0.958	0.730	0.890	0.885	0.800
C4.5	0.784	0.73–0.836	0.816	0.903	0.619	0.844	0.736	0.672
Logistic regression	0.769	0.719–0.825	0.841	0.951	0.587	0.840	0.841	0.692
Random forest	0.747	0.695–0.800	0.826	0.986	0.460	0.806	0.935	0.617
Bayes	0.734	0.678–0.785	0.797	0.896	0.571	0.827	0.706	0.632
GRACE	0.729	0.665–0.788	0.609	0.507	0.841	0.880	0.427	0.360
SVM	0.723	0.671–0.773	0.826	0.986	0.460	0.807	0.935	0.617
BP neural network	0.723	0.67–0.779	0.807	0.938	0.508	0.813	0.780	0.615
NEWS	0.687	0.622–0.753	0.556	0.431	0.841	0.861	0.393	0.143
MEWS	0.673	0.605–0.736	0.676	0.924	0.365	0.769	0.676	0.474
KNN	0.616	0.751–0.658	0.763	0.993	0.238	0.749	0.938	0.380

Abbreviations: BP, back propagation neural network; GRACE, Global Registry of Acute Coronary Events; KNN:K‐nearest neighbor; LR, logistic regression; MEWS, Modified Early Warning Score; NEWS, National Early Warning Score; NPV, negative predictive value; PPV, positive predictive value; SVM, support vector machine.

After considering these scores, especially the AUC value, we chose XGboost as the final prediction model. XGBoost is a boosting tree method in which each decision tree can be drawn, as shown in Figure S2.

### 3.4 Comparison with existing risk prediction models

We next compared our prediction models with three commonly cited risk prediction models for ACS patients: GRACE, NEWS, and MEWS. Except the KNN model, all other models produced a significant improvement of the outcome prediction compared with NEWS and MEWS, which had AUCs of 0.687 (95%CI: 0.622–0.753) and 0.673 (95%CI: 0.605–0.736), respectively (Figure S1B). However, three machine learning models ‐ SVM, BP neural network, and KNN ‐ had a worse performance than GRACE, which had an AUC of 0.723 (95%CI: 0.659–0.78) (Table 2).

## 4 DISCUSSION

In the present study, we used multivariate clinical features to develop eight machine learning models to predict cardiac arrest 24 h prior to the event in ACS patients. We found that most of these eight machine learning models improved prediction performance compared with three commonly used risk prediction models ‐ GRACE, NEWS, and MEWS. We also found that the XGBoost algorithm was the most accurate compared to the other seven other algorithms, and showed promising discrimination for detecting in‐hospital cardiac arrest. Moreover, we visualized the XGBoost model, which provided face validity for clinicians who are interested in using this flexible algorithm.

The XGBoost model has been extensively used in a variety of data‐mining fields for regression and classification due to its impressive accuracy and usability,^20^, ^21^, ^22^ although, there is currently less literature describing the use of XGBoost for predicting cardiac arrest. In our study, the XGBoost algorithm showed promising performance and had better prediction power compared to the other machine learning models, with an AUC value of 0.958, a specificity of 95.8%, and a sensitivity of 73%. The reasons for the high performance of the XGBoost model are as follows: (1) during training, the XGBoost algorithm generated a series of decision trees in a gradient boosting manner, and produced the next decision tree based on the current one to better predict the outcome; (2) after training, a risk prediction system composed of a series of decision trees was achieved; and (3) during application, the output predicted risk was the cumulative score of each decision tree, which indicates the likelihood of the predicted outcome. Therefore, the XGBoost model we generated can effectively stratify high risk ACS patients for cardiac arrest and truly assist clinicians with making appropriate treatment decisions. This prediction model will allow a monitoring alert system and life‐saving strategy to be implemented shortly before the occurrence of an adverse event.

In the field of predicting in‐hospital cardiac arrest in ACS patients, few studies have been performed to compare various machine learning algorithms. Churpek et al.^13^ conducted a study of machine learning methods for predicting clinical deterioration, including cardiac arrest, ICU transfer, and death. They reported that at 24 h prior to the occurrence, the random forest and XGBoost models achieved the most accurate prediction, with AUCs of 0.80 and 0.79, respectively. However, in that study, no other performance parameters were considered. Samaneh et al.^10^ developed a machine learning model to predict cardiac arrest for adult patients with sepsis, and showed that the random forest and XGBoost models generated the best values of accuracy and specificity. In addition, the XGBoost algorithm produced the best precision value, and the SVM model generated the highest sensitivity. However, the other criteria of these models were very low and unacceptable. Therefore, Samaneh et al. proposed a stacking machine learning model, which combined algorithms of the random forest, XGBoost, SVM, and logistic regression models, and finally obtained acceptable values for these criteria, with an AUC of 0.82, an accuracy of 0.76, a sensitivity of 0.77, a specificity of 0.76, and an F1 score of 0.31. A systematic review^11^ of the use of machine learning to predict cardiac arrest illustrated that (1) the SVM algorithm provided the best overall performance of AUC; (2) the KNN algorithm showed the best performance of specificity and accuracy; and (3) the BP neural network algorithm obtained a better sensitivity metric. Thus, there is no agreed upon best algorithm. The efficiency of a particular machine learning algorithm to predict cardiac arrest heavily depends on the population, samples, feature sets, and the ratio of cardiac arrest cases to normal patients. In this regard, comparing the outcomes of current work with those from previous studies is challenging.

Previously, Jeongmin et al.^23^ introduced feasible artificial intelligence with simple trajectories to predict adverse catastrophic events (FAST‐PACE), which consisted of simple vital signs, and found that FAST‐PACE outperformed MEWS and NEWS. In another study, Churpek and colleagues^13^ developed nine common machine learning models for ward deterioration in five hospitals, and showed that all models were more accurate than MEWS. In order to evaluate the effectiveness of the machine learning models we generated in this study, we compared them with three standard risk prediction systems ‐ GRACE, NEWS, and MEWS ‐ all of which were constructed by conventional methods. Except for the KNN model, all other machine learning models produced a significant improvement in prediction value compared to the two previous prediction models, NEWS and MEWS. To the best of our knowledge, this is the first study to compare machine learning models with GRACE for predicting cardiac arrest. The GRACE risk score was developed using a logistic regression approach, which was intended to predict in‐hospital mortality in the short‐and long term for ACS patients.^24^, ^25^ The most recent guidelines by international societies recommend that the GRACE risk score should be used in practice as a risk stratification tool.^26^, ^27^ In the present study, we found that the performance of GRACE was superior to the algorithms of SVM, BP neural network, and KNN. Thus, it appears that no one machine learning algorithm will be superior to traditional ones, and that no algorithm will be the most accurate in every scenario. Comparisons of algorithms in different research areas and datasets may yield different results.

This study has limitations that need to be addressed in future studies. First, the criticism of most machine learning algorithms is that they are black boxes. Although we derived an XGboost node graph, it was still unable to be applied in a straight forward manner, which in turn may make clinicians wary of its clinical application. Second, the prediction model generated in this study was established based on limited data obtained from a Chinese population and no external validation was performed. Therefore, the XGBoost model should be further evaluated using more data from other ethnic groups and regions in future studies.

## 5 CONCLUSION

In this study, we developed and evaluated the effectiveness of several machine learning algorithms for predicting cardiac arrest in ACS patients. We found that most of the algorithms, specifically the XGBoost algorithm, showed promising performance and had better power than the existing prediction systems, such as GRACE, NEWS, and MEWS. We suggest that the XGBoost model can be used as a complementary tool in medical decision‐making for early intervention and prevention of cardiac arrest in ACS patients.

## CONFLICT OF INTEREST

The author declares that there is no conflict of interest that could be perceived as prejudicing the impartiality of the research reported.

## ROLE OF THE SPONSORS

The sponsors played no role in this study. There was no industry involvement in any of the following: the design or conduct of the study; the collection, management, analysis, and interpretation of the data; the preparation, review, and approval of the manuscript; and the decision to submit the manuscript for publication.

## Supporting information


**Figure S1.** ROC of machine learning models, GRACE, NEWS, and MEWS predicting cardiac arrest. SVM: support vector machine, BP: back propagation neural network, KNN: K‐nearest neighbor.

**Figure S2.** XGBoost node graph. The inside nodes represent values for an attribute test and the leaf nodes with scores represent a decision of predicting cardiac arrest.
