# Supplementary material for: Machine learning for early prediction of in‐hospital cardiac arrest in patients with acute coronary syndromes
Source: Clin Cardiol. 2021 Feb 14;44(3):349–56. doi: 10.1002/clc.23541 (PMC7943901; doi:10.1002/clc.23541)
Supplement: Supplementary file 1 — Figure S1. ROC of machine learning models, GRACE, NEWS, and MEWS predicting cardiac arrest. SVM: support vector machine, BP: back propagation neural network, KNN: K‐nearest neighbor. [file CLC-44-349-s002.pdf]

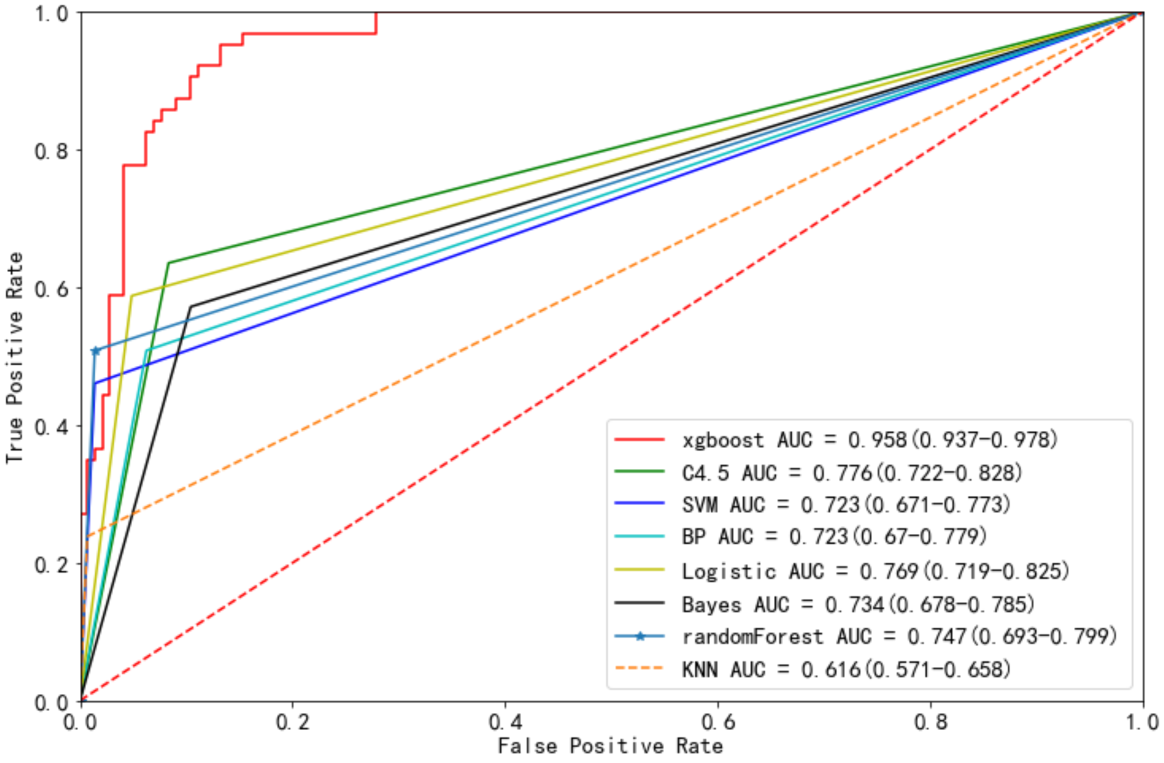

(A)

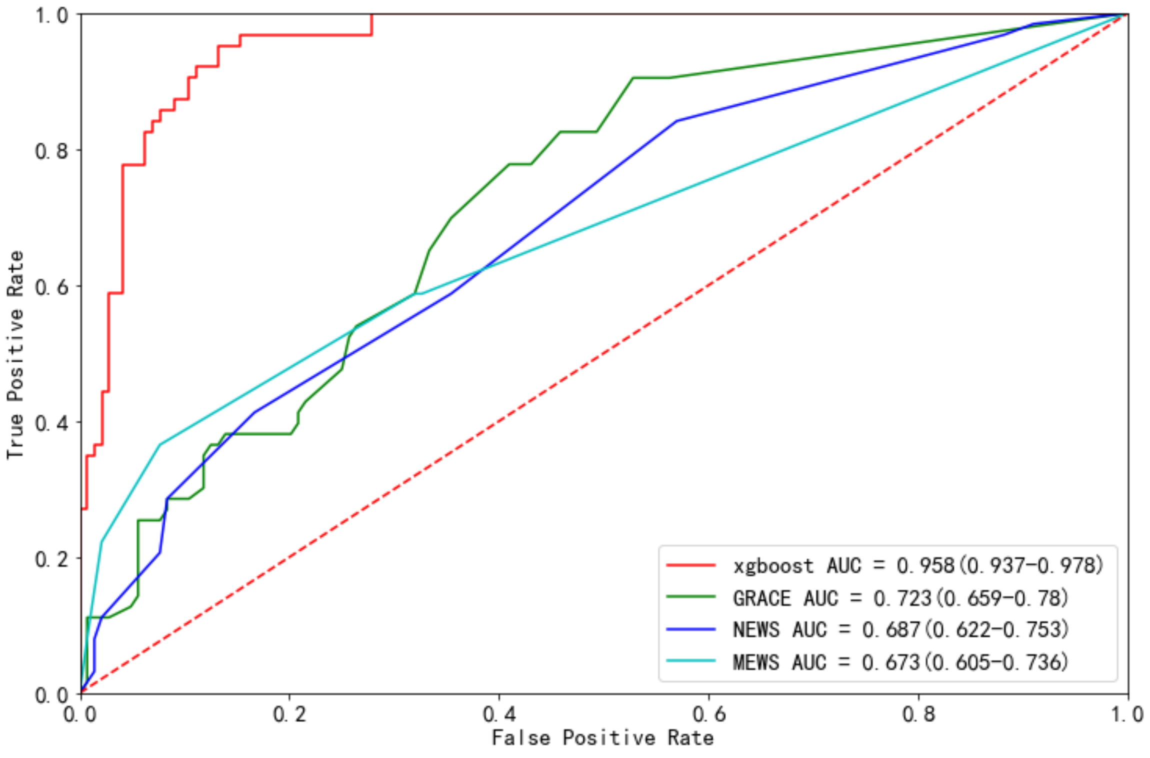

(B)

Supplementary figure.1 ROC of machine learning models, GRACE, NEWS, and MEWS predicting cardiac arrest

SVM: support vector machine, BP: back propagation neural network, KNN: K- nearest neighbor
