# Supplementary material for: Machine learning for early prediction of in‐hospital cardiac arrest in patients with acute coronary syndromes
Source: Clin Cardiol. 2021 Feb 14;44(3):349–56. doi: 10.1002/clc.23541 (PMC7943901; doi:10.1002/clc.23541)
Supplement: Supplementary file 2 — Figure S2. XGBoost node graph. The inside nodes represent values for an attribute test and the leaf nodes with scores represent a decision of predicting cardiac arrest. [file CLC-44-349-s001.pdf]

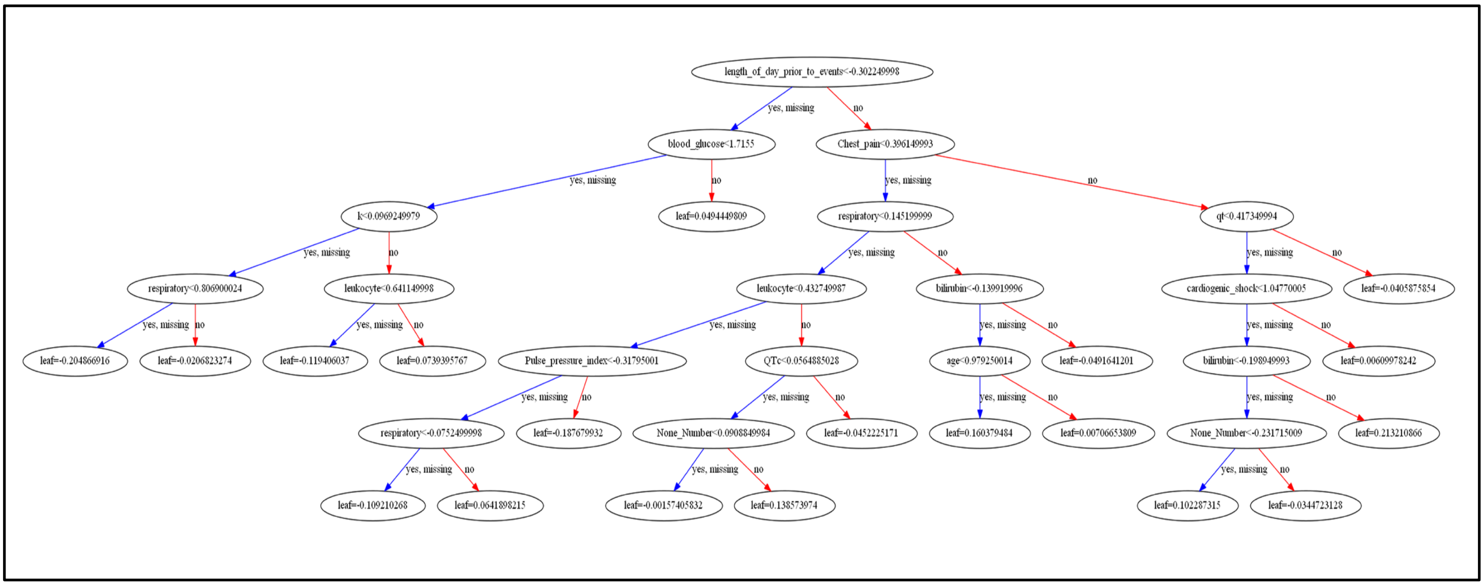

Supplementary figure.2 XGBoost node graph

The inside nodes represent values for an attribute test and the leaf nodes with scores represent a decision of predicting cardiac arrest
